# Nutritional considerations for gender-diverse people: a qualitative mini review

**DOI:** 10.3389/fnut.2024.1332953

**Published:** 2024-02-14

**Authors:** Em Jun Eng Mittertreiner, Abbey Hunter, Emilie Lacroix

**Affiliations:** ^1^Maritime Eating and Appearance Lab, Department of Psychology, University of New Brunswick, Fredericton, NB, Canada; ^2^School of Education, St. Thomas University, Fredericton, NB, Canada

**Keywords:** eating disorders, gender diversity, transgender, gender-affirming healthcare, nutrition, review, disordered eating, qualitative

## Abstract

Dietitians working with gender-diverse people may require different skills and knowledge than those caring for cisgender men and women, as indicated by a growing body of literature that highlights gender-diverse people's unique experiences with and relationships to nutrition and eating behaviors. To provide insight into how dietitians can best serve this population, this mini review identifies and summarizes qualitative studies that investigate gender-diverse people's lived experiences and perspectives regarding nutrition, eating disorders, and access to eating-related healthcare services. Fourteen studies examining nutrition or eating behaviors among gender-diverse samples were selected through a systematic search and screening process: 11 focused on disordered eating or eating disorders and the remaining three focused on nutritional needs, nutritional knowledge, and food insecurity. Extracted themes included: using dietary restriction to suppress secondary sex characteristics or conform to societal norms; the impact of gender-affirming care on disordered eating; negative experiences with, and beliefs about, nutrition and eating disorders healthcare services; and suggestions for clinicians. Recommendations discuss the need for increased trans literacy among clinicians, the creation of safe spaces for gender-diverse people with eating disorders, and the importance of dual competencies in eating disorders treatment and gender-affirming care.

## 1 Introduction

Gender and sex are important factors to consider when determining an individual's nutritional needs. Traditionally, dietetics literature has primarily examined binary sex differences between females and males. However, more studies are emerging that emphasize the interplay between nutritional considerations and gender-diverse (i.e., non-cisgender; does not identify with birth sex) identities. For example, some non-cisgender people pursue hormone therapy to masculinize or feminize their secondary sex characteristics. Hormone therapies can affect a person's caloric needs, alter their hunger and satiety cues, and increase their risk for nutrition-related diseases (1). Thus, special attention is required when monitoring the nutritional needs of people undergoing hormone replacement therapy, as dietary requirements may shift over time (1).

Gender-diverse individuals' relationships to nutrition also differ in that they are at greater risk of disordered eating, for several reasons (2). First, disordered eating can emerge at the onset of puberty to modulate the development of secondary sex characteristics (3). Second, eating disorders can serve as a coping mechanism, arising in response to stressors commonly faced by gender-diverse people, such as bullying, transphobia, and healthcare barriers (4). Third, disordered eating can be triggered when people are encouraged to gain or lose weight to be eligible for gender-affirming medical procedures (5). Notably, many trans men and non-binary people assigned female at birth (AFAB) are denied chest masculinization surgery due to BMI limits set by surgeons (5). When considerations such as these are not accounted for, nutritional care—particularly eating disorders treatment—may be less effective or even iatrogenic (6). In fact, several qualitative studies have reported that the efficacy and acceptability of gender-diverse participants' eating disorders treatment experiences hinged on whether their gender identity was considered during recovery [e.g., (3)].

A small but increasing number of qualitative studies are examining gender-diverse peoples' relationships to nutrition, eating disorders, and related healthcare services. The aim of this mini review is to synthesize qualitative literature in this area and generate recommendations as a basis for best-practice guidelines in the nutritional care of this population.

## 2 Methods

This mini review used thematic synthesis methodology (7). On June 1, 2023, we conducted a systematic search of Embase, Medline, CINAHL, and PsycInfo. We used keywords related to gender diversity, nutrition and diet, and qualitative research (Supplementary material). No date or language limits were set. Included studies met the following a priori criteria: (1) published in a peer-reviewed journal, (2) featured empirical qualitative primary research, (3) involved a sample of gender-diverse people, and (4) explored experiences with nutrition, diet, or eating behaviors. Quantitative studies, gray literature, reviews, and commentaries were excluded. Studies that recruited broadly from the 2SLGBTQIA+ population and studies of binary gender/sex differences were not included unless analyses related to gender diversity were explicitly discussed.

After deduplication, the systematic searches identified 2,844 unique records, which were imported into Covidence for screening. E.J.E.M. and A.H. screened studies at the title/abstract level (*k* = 0.63; agreement = 99.1%), retaining 23 studies. E.J.E.M. conducted the full-text screening, ultimately selecting 14 studies for inclusion (Supplementary Tables 1, 2).

### 2.1 Generating codes and themes using thematic synthesis

To enhance familiarity with the material and facilitate the coding process, the first author read each study and extracted all participant quotes related to gender diversity and nutrition using NVivo. Free line-by-line coding was used to label each quote according to its semantic meaning and content. Then, the codes were inspected for similarities and differences, and grouped accordingly into narrow descriptive categories or sub-themes. These categories were then organized into broader analytical themes based on conceptual similarities. Because the objective was to accurately represent participants' perspectives rather than critically analyze or challenge them, an experiential and realist lens was used; this is in contrast to a critical or constructivist framework that might have focused more on interrogating or unpacking the latent meanings underlying participants' sentiments (8).

### 2.2 Trustworthiness of analyses

As direct member-checking was not possible due to secondary data analysis, the primary author consulted with their colleague, M.M., a trans man with lived experience of an eating disorder who works as a peer support coordinator for trans youth at a large eating disorders nonprofit organization. M.M. provided essential feedback on the codes and themes developed by E.J.E.M., confirming that the analyses were congruent with the experiential knowledge M.M. has accumulated while supporting trans people with eating disorders.

### 2.3 Research team and reflexivity

All authors are members of the Maritime Eating and Appearance Lab (MEAL) at the University of New Brunswick, which specializes in understanding the development, prevention, and treatment of eating disorders and body image concerns. The primary author, E.J.E.M., is a mixed-race, queer, and disabled transmasculine non-binary graduate student in clinical psychology who has undergone training in anti-oppressive and feminist qualitative research. As a transgender young person with both positive and negative experiences of inpatient eating disorders treatment, they wrote self-reflexive journal entries throughout the data analysis process to understand how their positionality and lived experiences might bias the lens through which they viewed and coded the data. The supervising author, E.L., is a White, cisgender, heterosexual woman with a Ph.D. in Clinical Psychology, specializing in eating disorders research. She identifies as an ally to the 2SLGBTQIA+ community and employs a weight- and gender-inclusive lens in her research and clinical activities. A.H., a MEAL research assistant, is a queer, cisgender post-baccalaureate non-graduate student in Education. She has undergone training in feminist research, with a focus on the lived experiences of 2SLGBTQIA+ individuals, and identifies as an ally to the 2SLGBTQIA+ community.

## 3 Results

This mini review includes 264 quotes from 14 total studies (Supplementary Table 3). The quotes are divided into nine main themes with 30 sub-themes. As not all themes could be fully covered in this section, only the five most well-represented sub-themes (i.e., those with the most supporting articles) and their respective main themes are discussed. As studies did not consistently specify participants' pronouns, gender-neutral pronouns (i.e., they/them/theirs) will be used to refer to participants.

### 3.1 Restrictive nutrition as a “DIY” solution to gender affirmation

Thirty-three quotes from 10 articles related to ways participants sought feelings of gender affirmation through manipulating their dietary intake, often in the absence of access to gender-affirming medical care.

#### 3.1.1 Manipulating secondary sex characteristics

A commonly cited rationale for dietary restriction was the potential for weight loss to suppress unwanted, gendered body features, such as the appearance of breasts or the distribution of fat around the hips and stomach. Many gender-diverse individuals hoped that they could “…diet [their] gender away completely” [(9), p. 306]. Interestingly, it was rare for trans feminine people to report eating more to accentuate curves, or for trans masculine people to report eating more to build muscle. Instead, most people took a restrictive approach regardless of identity, suggesting they viewed thinness as a hallmark of being both “feminine enough” and “masculine enough.”

One trans masculine participant discussed how “weight gain would have brought forth my feminine figure, which was disgusting to me” [(9), p. 306]. Another was driven to achieve “crazy weight loss” so that their “curves would disappear” [(9), p. 306]. Meanwhile, a trans feminine participant commented that they “…had anorexia as a teenager as an attempt to stunt my growth and avoid growing muscle mass” [(10), p. 6]. Many resorted to dietary restriction in response to the inaccessibility of gender-affirming healthcare: “…my eating disorder started as a way to stop [menstruation] and breast development, so access to binders, hormone blockers/HRT and gender-affirming [are] would have helped me more when I was younger than therapy surrounding body image” [(11), p. 4]. Similarly, another discussed how they “…developed bulimia when I was a teenager as a way to slow down puberty since I was denied [gender-affirming] medical care” [(10), p. 6]. Overall, it appears common for gender-diverse people to use risky methods of shape and weight manipulation as a tactic to suppress or accentuate secondary sex characteristics when safe, regulated gender-affirming care is not accessible.

### 3.2 Disordered eating to conform with social and gender norms

Thirty-four quotes from eight articles highlighted how a desire to conform to social norms—whether for emotional, interpersonal, or physical safety reasons—can influence gender-diverse people's relationships with nutrition.

#### 3.2.1 Achieving normative gender ideals

People across gender identities felt pressure to conform to binary gender norms; for example, one trans woman commented, “I want to fit into American cultural ideals about what a pretty woman looks like—maybe my face is masculine, but at least I can be thin” [(10), p. 5]. Another trans feminine person discussed how “…size is gendered…the thinner you are, the more feminine…[and] I wanted to be read more as female” [(6), p. 6]. Non-binary people also felt pressure to be thin, with one participant discussing how “to be androgynous and to be taken seriously as Nonbinary…you had to be thin” [(10), p. 7]. Another participant brought up how intersecting female body ideals and weight stigma led them to wish for a smaller body, believing that “I would be treated differently every day if I was smaller” [(6), p. 6]. Some participants took an opposite perspective, with one noting that, “…throughout recovery, there was that systemic piece of looking at gender roles and sexuality and…diet culture…I stopped really trying to fit into the boxes [of gender], and it's been a critical piece of my recovery” [(6), p. 8]. This quote, and others, demonstrate resistance to conformity and an awareness of larger socio-political forces at play. Social norms clearly played different roles for different participants, with some embracing conformity to feel affirmed in their gender and others feeling empowered to eschew social norms entirely.

### 3.3 Gender affirmation and ED recovery

Thirty-six quotes from eight articles underscored how taking steps toward social and medical transition can change one's relationship to food, and reciprocally, how recovering from disordered eating can influence gender dysphoria.

#### 3.3.1 Gender-affirming changes leading to improved nutrition and self-care

For many, receiving gender-affirming care (e.g., hormone therapy, surgery) or taking steps toward social affirmation (e.g., legal name change, exploring new hair and clothing styles) reduced disordered eating patterns. One person commented, “…when I started transitioning and moving toward HRT, I felt less distressed with my body and managed to gain weight to a healthy level” [(10), p. 5]. Another described their transition as “just totally joyful for me the whole time” [(12), p. 56]; despite noticing some unwanted changes with HRT such as weight gain, they were “not concerned about it at all because it's like, I have a body that I inhabit, that's fucking cool” [(12), p. 56].

For one participant, starting testosterone “made me want to take better care of my body because I'm getting the body that I actually want to have so I want to take care of it” [(13), p. 154]. Another trans masculine person no longer felt like weight loss was required to pass as a man, despite having gained weight on their hips due to testosterone: “…I have more curves. But it's not as bad because I'm tall and hairy and I have a low voice” [(13), p. 154]. Increased self-esteem was also a common theme, with a post-surgery participant stating that “I feel like I am myself now, even with my fat. Maybe I've gained some kind of self-confidence” [(9), p. 308]. These quotes suggest that taking steps toward gender affirmation helped empower participants with feelings of care, responsibility, and motivation, encouraging them to better nurture their bodies.

### 3.4 Unique challenges faced by gender-diverse people

Forty-three quotes from eight articles discussed difficulties that gender-diverse people encounter when seeking to improve their relationships with food, such as a paucity of safe spaces, poor awareness that gender-diverse people can experience disordered eating, and the fact that gender-diverse people's ED recovery needs may diverge from what treatment programs typically offer.

#### 3.4.1 Trans-specific issues in ED recovery

According to one participant, “the needs [in eating disorder care] are quite different in the case of trans people” [(3), p. 395]. Multiple articles specifically identified body-positive approaches as inappropriate for those with gender dysphoria. One person worried that “…the issues around my trans-specific body discomfort will be ignored or downplayed in favor of the traditional ‘learn to love your body' approach to treatment” [(14), p. 140]. Another emphasized how approaches to treating gender dysphoria are discordant with those for EDs: “…the prescription for treating body dysphoria is to change your body, right? Take hormones. Get surgery…But the prescription for [an] ED…is the exact opposite: love yourself as you are, and don't change a thing. It is a Catch-22” [(6), p. 9]. Others found that treatment settings overlooked the nuanced connection between gender and EDs: “[Gender identity] is a multifaceted thing…[Treatment] just sort of missed the complexity of all those pieces” [(6), p. 11]. Another participant similarly expressed that treatment missed the mark, feeling “like the odd one out because my eating disorder wasn't about diet culture. It was about gender issues and trauma” [(3), p. 392]. Considering these experiences, it is concerning that mainstream treatment approaches do not typically address the types of gender-related trauma or body image concerns prevalent among gender-diverse people.

### 3.5 Experiences with care providers

Fifty quotes from nine articles discussed gender-diverse people's beliefs and experiences regarding eating-related healthcare. Few recounted positive, gender-affirming clinician interactions, while many discussed having their gender identity erased by dietitians or therapists, avoiding nutrition-related healthcare due to fears of transphobia, having to educate providers while enduring insensitive comments, or feeling misunderstood due to poor trans literacy among providers. Some had specific recommendations for clinicians or organizations looking to improve services for gender-diverse people.

#### 3.5.1 Recommendations for care providers

One participant discussed avoiding their university's nutrition counseling services due to worry that they would “not understand that my eating disorder stems from gender dysphoria” [(15), p. 37], while another wished for “…nutrition advice…for people who don't have binary hormone systems (do I follow men's advice? Women's?)” [(11), p. 4]. Another participant found it “nerve-wracking” to “…wonder whether a program which works with women means that it works with all women, or only cis women,” and suggested it would be “reassuring” for healthcare services' promotional materials to make “…some sort of reference to their inclusivity of transgender/gender-diverse people” [(14), p. 143]. Further, a participant recommended that ED-focused programs hoping to support gender-diverse clientele “…give presentations within other groups, to make known your services” and “…reach out to LGBT centers…consider helping them establish eating-disorder support groups” [(14), p. 143]. One participant requested that clinicians “…always ask for pronouns. Use the name they ask you to use—I can't stress this enough” [(14), p. 143]. Many suggested that clinicians engage in trans literacy training: “I need current ED…health providers to understand transness…All of these programs advertise themselves to trans people as being trans competent…but then the programs are not very trans competent when you get there” [(11), p. 4].

## 4 Discussion

This mini review examined qualitative studies related to gender diversity and nutrition to derive actionable recommendations for dietitians, other healthcare providers, and healthcare organizations. Themes included: using dietary restriction to suppress secondary sex characteristics or conform to societal norms; the impact of gender-affirming care on disordered eating; negative experiences with—and beliefs about—nutritional and eating disorders healthcare services; and recommendations for clinicians. These themes can be traced back to one overarching issue: gender-diverse people experience disproportionate barriers to accessing trans-competent dietetics services, especially services focused on treating eating disorders. As such, these recommendations will focus on practices that dietitians and ED-focused services can adopt to facilitate more accessible and accepting healthcare environments for gender-diverse individuals.

Multiple participants described negative encounters with dietitians or other nutritional services that were not trans-competent, and others expressed avoiding nutritional services entirely due to fears of transphobia and insensitive providers. This suggests two calls to action: first, nutrition-focused service providers must engage in trans literacy training, and second, service providers who are trans-literate should be explicit about their openness to gender-diverse clients via their promotional materials. For example, if descriptions of each staff member are listed on a provider's website, pronouns should be included, and any specialized experience with gender-diverse clientele should be noted. For in-person services, availability of gender-neutral washrooms is also an important detail. Providing this information up front could address prospective clients' reservations and make clients more comfortable disclosing their trans identity to providers. Clients who feel unable to disclose their gender identity in healthcare settings can experience intense worry, hypervigilance, and self-consciousness (16), which may limit providers' abilities to build rapport. Thoughtfully constructed intake forms are another administrative gesture that can signal safety. The “sex” sections of such forms can be improved through increased acknowledgment of intersex identities [i.e., including an “intersex” or “other (specify)” option along with man/male and woman/female], while the “gender” sections could benefit from a “write-in” option for those with diverse gender identities. Additionally, intake forms should ask for clients' affirmed names and pronouns (17). Finally, some participants wished for more gender-diverse clinicians. Hiring gender-diverse clinicians would provide clients with more effective support *and* create more diversity in the dietetics field.

### 4.1 Addressing eating disorders in gender-diverse individuals

Eleven of the 14 studies focused on disordered eating among gender-diverse individuals. Given that gender-diverse people are up to eight times more likely to engage in disordered eating (18), it is important that clinicians understand how gender diversity impacts eating behaviors, and that they integrate this understanding into assessment, case conceptualization, and treatment. To this end, we provide recommendations in each of these areas.

#### 4.1.1 Understanding

Understanding and empathizing with gender-diverse clients' lived experiences is key to bringing gender-affirming practices into ED care. Participants suggested that clinicians start by attending events in the local 2SLGBTQIA+ community, reading fiction books and autobiographies by gender-diverse people, and participating in rallies supporting policies related to gender diversity (14). Additionally, dietetics or ED services can partner with 2SLGBTQIA+ resource centers or programs (14), allowing for the creation of support groups coordinated by a team of practitioners: some with expertise in gender diversity, and others with specialized nutritional or ED knowledge.

#### 4.1.2 Assessment and case conceptualization

One participant stated that they were never asked about gender identity/dysphoria while in ED treatment, referred to this as a “HUGE oversight” [(11), p. 4]. As such, nutritional and ED assessments should routinely inquire about gender identity and possible dysphoria. In terms of case conceptualization, dietitians who work in the eating disorders field must be equipped with trans-specific competencies, such as understanding how gender dysphoria interacts with disordered eating (3). Participants discussed how their relationships to nutrition and eating were influenced by pressures to conform to social and gender norms; these norms varied based on participants' perceptions of the societal ideal for the gender they identified with. Clinicians working in the ED field should be cognizant of gendered stereotypes (i.e., the idea that women feel pressure to be thin and men feel pressure to be muscular) and allow clients to explore how pressures to socially conform have uniquely affected them in the context of their gender identity. ED dietitians should equip clients with tools to understand how their nutritional habits and body ideals are shaped by gender norms and expectations, which may give clients more clarity regarding whether their personal “ideal” gender presentation stems from a genuine drive to be true to oneself, or an external pressure to match a certain body norm.

#### 4.1.3 Treatment planning

Participants expressed that traditional approaches to treating eating disorders such as “learning to love your body” can be ineffective for gender dysphoric people, especially if gender-affirming therapy is not accessible (3, 11, 14). Body neutrality may be a more realistic goal for this population. Further, participants identified gender-affirming care as essential to their ED recovery journeys. Dietitians should be prepared to assist clients in transition-related matters, such as helping those on hormonal therapies navigate changing appetites and metabolisms (11) and adhere to the rigorous nutritional guidelines prescribed for gender-affirming surgeries (5). Thus, gender identity and gender dysphoria should be explicitly addressed at the assessment and case conceptualization stages, and consideration of gender-affirming medical care should be a critical component of treatment plans for gender-diverse clients.

## 5 Conclusion

In sum, this article took a participant-centered approach to understanding gender-diverse people's relationships to nutrition by analyzing 264 quotes from 14 qualitative studies. Participants highlighted the difficulties experienced by gender-diverse people attempting to access nutritional care, underscoring the need to improve trans literacy among healthcare providers. This is especially true for those who work in the field of eating disorders, given the high prevalence of disordered eating demonstrated within these 14 studies. We recommend that future studies more fully explore gender-diverse people's needs and preferences when it comes to eating disorders treatment and support, and that clinicians prioritize gender considerations at the stages of assessment, case conceptualization, and treatment planning. Further, it should be noted that all studies included specifically set out to study gender-diverse people. We recommend that more studies in the general ED field collect data regarding gender diversity and ensure that participation is accessible to gender-diverse participants through inclusive sampling methodologies (e.g., not excluding non-binary people simply to avoid low cell counts) and inclusive data collection strategies (e.g., ensuring that demographics questions allow for participants to indicate gender-diverse identities).

## Author contributions

EJEM: Conceptualization, Data curation, Formal analysis, Investigation, Methodology, Writing—original draft. AH: Data curation, Writing—review & editing. EL: Conceptualization, Writing—review & editing, Data curation, Supervision.
