# Supplementary material for: Nutritional considerations for gender-diverse people: a qualitative mini review
Source: Front Nutr. 2024 Feb 14;11:1332953. doi: 10.3389/fnut.2024.1332953 (PMC10899494; doi:10.3389/fnut.2024.1332953)
Supplement: Supplementary file 1 [file Table_1.DOCX]

**Search Strategies**

**OVID MEDLINE**

1 exp Gender Identity/ or exp Sex Reassignment Procedures/ or exp "Sexual and Gender Minorities"/ or "Health Services for Transgender Persons"/ or Transsexualism/ or Gender Dysphoria/ or "Sexual and Gender Disorders"/ or Transvestism/

2 ((gender adj1 (identi* or self-concept? or reassignment or therapy or clinic? or affirm* or service? or minorit* or dysphori? or nonconform* or varian* or queer or spectrum? or divers* or role? or incongruen* or congruen* or atypical* or norm* or non-norm* or expression* or creativ* or fluid or transition)) or (trans adj1 (boy? or girl? or man or woman or men or women or male? or female? or masculine or feminine)) or (transgender* or transmasculine or transfeminine or transmale? or transfemale? or transm#n or transwom#n or transsex* or nonbinary or genderdivers* or genderqueer or agender or genderfluid or ((hormon* or testosterone or estrogen) adj 2 therapy))).mp.

3 exp "Feeding and Eating Disorders"/ or exp Eating/ or exp Diet/ or exp Feeding Behavior/ or exp Nutritional Requirements/ or Nutritional Status/ or exp Food Supply/ or exp "Diet, Food and Nutrition"/ or Nutrition Assessment/ or Nutrition Policy/ or exp Nutrition Surveys/ or exp Nutrition Therapy/ or exp Body Weight/ or exp "Nutritional and Metabolic Diseases"/

4 (anorexi* or bulimi* or ((binge or binging or purge or purging) adj2 disorder*) or ((eating or feeding or food) adj2 (disorder* or patholog*)) or nutri* or diet* or eating or weight or bmi or body mass index or calorie?).mp.

5 Exp Qualitative Research/ or Grounded Theory/ or Community-Based Participatory Research/ or qualitative.mp.

6 (1 or 2) and (3 or 4) and 5

**OVID EMBASE**

1 exp Gender Diversity/ or exp "Transgender and Gender Nonbinary"/ or exp "Sexual and Gender Minority"/ or femininity/ or masculinity/ or exp Gender Identity/ or exp Gender Dysphoria/ or Transsexuality/ or Sex Reassignment/ or Sex Role/ or Cross-Dressing/ or Transphobia/

2 ((gender adj1 (identi* or self-concept? or reassignment or therapy or clinic? or affirm* or service? or minorit* or dysphori? or nonconform* or varian* or queer or spectrum? or divers* or role? or incongruen* or congruen* or atypical* or norm* or non-norm* or expression* or creativ* or fluid or transition)) or (trans adj1 (boy? or girl? or man or woman or men or women or male? or female? or masculine or feminine)) or (transgender* or transmasculine or transfeminine or transmale? or transfemale? or transm#n or transwom#n or transsex* or nonbinary or genderdivers* or genderqueer or agender or genderfluid or ((hormon* or testosterone or estrogen) adj 2 therapy))).mp.

3 exp nutrition/ or exp food insecurity/ or exp eating disorder/ or exp nutritional disorder/ or exp body weight/ or exp body weight disorder/

4 (anorexi* or bulimi* or ((binge or binging or purge or purging) adj2 disorder*) or ((eating or feeding or food) adj2 (disorder* or patholog*)) or nutri* or diet* or eating or weight or bmi or body mass index or calorie?).mp.

5 exp qualitative research/ or exp practice guideline/ or grounded theory/ or exp participatory research/ or qualitative analysis/ or qualitative.mp.

6 (1 or 2) and (3 or 4) and 5

**EBSCO PsycInfo**

S1 DE "Gender Dysphoria" OR DE "Gender Nonconforming" OR DE "Gender Transition" OR DE "Gender Nonbinary" OR DE "Gender Expression" OR DE "Gender Reassignment" OR DE "Gender Identity" OR DE "Gender Nonbinary" OR DE "Gender Nonconforming" OR DE "Gender Affirming Care" OR DE "Hormone Therapy" OR DE "Personal Pronouns" OR DE "Transsexualism" OR DE "Transgender" OR DE "Transvestism" OR DE "Intersex" OR DE "Affirmative Therapy"

S2 TI (gender W1 (identi* OR self-concept OR reassignment OR therapy OR clinic OR service OR minority OR dysphori#? OR nonconform* OR varian* OR queer OR spectrum OR diver* OR role OR incongruen* OR congruen* OR atypical* OR norm* OR non-norm* OR expression OR creativ* OR fluid OR transition)) OR AB (gender W1 (identi* OR self-concept OR reassignment OR therapy OR clinic OR service OR minority OR dysphori#? OR nonconform* OR varian* OR queer OR spectrum OR diver* OR role OR incongruen* OR congruen* OR atypical* OR norm* OR non-norm* OR expression OR creativ* OR fluid OR transition))

S3 TI (trans W1 (boy OR girl OR man OR woman OR male OR female OR masculine OR feminine)) OR AB (trans W1 (boy OR girl OR man OR woman OR male OR female OR masculine OR feminine))

S4 TI (transgender* OR transmasculine OR transfeminine OR transmale* OR transfemale* OR transm?n OR transwom?n OR transsex* OR transvest* OR nonbinary OR non-binary OR genderdiver* OR genderqueer*) OR AB (transgender* OR transmasculine OR transfeminine OR transmale* OR transfemale* OR transm?n OR transwom?n OR transsex* OR transvest* OR nonbinary OR non-binary OR genderdiver* OR genderqueer*)

S5 S1 OR S2 OR S3 OR S4

S6 DE "Eating Behavior" OR DE "Binge Eating" OR DE "Dietary Restraint" OR DE "Emotional Eating" OR DE "Food Refusal" OR DE "Healthy Eating" OR DE "Binge Eating" OR DE "Healthy Eating" OR DE "Eating Attitudes" OR DE "Eating Disorders" OR DE "Anorexia Nervosa" OR DE "Avoidant/Restrictive Food Intake Disorder" OR DE "Binge Eating Disorder" OR DE "Bulimia" OR DE "Feeding Disorders" OR DE "Orthorexia" OR DE "Pica" OR DE "Purging (Eating Disorders)" OR DE "Rumination (Eating)" OR DE "Nutrition" OR DE "Calories" OR DE "Dietary Supplements" OR DE "Diets" OR DE "Food" OR DE "Mealtimes" OR DE "Weight Control" OR DE "Vitamins" OR DE "Obesity" OR DE "Nutritional Deficiencies"

S7 TI (((anorexi* OR bulimi* OR bing* OR purg*) W1 disorder*) OR ((eating OR feeding OR food) W1 (disorder* OR patholog*)) OR nutri* OR diet* OR eating OR weight OR bmi OR body mass index OR calorie*) OR AB (((anorexi* OR bulimi* OR bing* OR purg*) W1 disorder*) OR ((eating OR feeding OR food) W1 (disorder* OR patholog*)) OR nutri* OR diet* OR eating OR weight OR bmi OR body mass index OR calorie*)

S8 S6 OR S7

S9 DE "Qualitative Methods" OR DE "Focus Group" OR DE "Grounded Theory" OR DE "Interpretative Phenomenological Analysis" OR DE "Narrative Analysis" OR DE "Semi-Structured Interview" OR DE "Thematic Analysis" OR DE "Thematic Analysis" OR DE "Narrative Analysis" OR DE "Mixed Methods Research" OR DE "Item Analysis (Test)" OR DE "Differential Item Functioning" OR DE "Interpretative Phenomenological Analysis" OR DE "Grounded Theory" OR DE "Focus Group" OR DE "Content Analysis" OR DE "Digital Content Analysis" OR DE "Discourse Analysis" OR DE "Narrative Analysis" OR DE "Sentiment Analysis" OR DE "Social Network Analysis" OR DE "Thematic Analysis"

S10 TI qualitative OR AB qualitative

S11 S9 OR S10

S12 S5 AND S8 AND S11

**EBSCO CINAHL**

S1 (MH "Gender-Nonconforming Persons+") OR (MH "Gender Affirmation Procedures+") OR (MH "Gender Affirming Care") OR (MH "Gender Affirmation Surgery") OR (MH "Sexual and Gender Minorities+") OR (MH "Gender Transition") OR (MH "Gender Identity+") OR (MH "Gender Dysphoria") OR (MH "Nonbinary Persons") OR (MH "Transgender Persons+") OR (MH "Transphobia") OR (MH "Trans Women") OR (MH "Trans Men")

S2 TI (gender W1 (identi* OR self-concept OR reassignment OR therapy OR clinic OR service OR minority OR dysphori#? OR nonconform* OR varian* OR queer OR spectrum OR diver* OR role OR incongruen* OR congruen* OR atypical* OR norm* OR non-norm* OR expression OR creativ* OR fluid OR transition)) OR AB (gender W1 (identi* OR self-concept OR reassignment OR therapy OR clinic OR service OR minority OR dysphori#? OR nonconform* OR varian* OR queer OR spectrum OR diver* OR role OR incongruen* OR congruen* OR atypical* OR norm* OR non-norm* OR expression OR creativ* OR fluid OR transition))

S3 TI (trans W1 (boy OR girl OR man OR woman OR male OR female OR masculine OR feminine)) OR AB (trans W1 (boy OR girl OR man OR woman OR male OR female OR masculine OR feminine))

S4 TI (transgender* OR transmasculine OR transfeminine OR transmale* OR transfemale* OR transm?n OR transwom?n OR transsex* OR transvest* OR nonbinary OR non-binary OR genderdiver* OR genderqueer*) OR AB (transgender* OR transmasculine OR transfeminine OR transmale* OR transfemale* OR transm?n OR transwom?n OR transsex* OR transvest* OR nonbinary OR non-binary OR genderdiver* OR genderqueer*)

S5 S1 OR S2 OR S3 OR S4

S6 (MH "Eating Behavior+") OR (MH "Eating Disorders+") OR (MH "Night Eating Syndrome") OR (MH "Binge Eating Disorder") OR (MH "Food Habits") OR (MH "Food Fussiness") OR (MH "Orthorexia Nervosa") OR (MH "Bulimia Nervosa") OR (MH "Bulimia") OR (MH "Avoidant Restrictive Food Intake Disorder") OR (MH "Nutrition+") OR (MH "Nutrition Policy+") OR (MH "Nutrition Disorders+") OR (MH "Public Health Nutrition") OR (MH "Nutrition Services+") OR (MH "Nutrition Education") OR (MH "Nutritional Support Team") OR (MH "Nutritional Support+") OR (MH "Nutritional Assessment") OR (MH "Academy of Nutrition and Dietetics") OR (MH "Diet Therapy+") OR (MH "Nutritive Value+") OR (MH "Nutritional Counseling") OR (MH "Nutritional Status") OR (MH "Dietary Supplementation") OR (MH "Undernutrition")

S7 TI (((anorexi* OR bulimi* OR bing* OR purg*) W1 disorder*) OR ((eating OR feeding OR food) W1 (disorder* OR patholog*)) OR nutri* OR diet* OR eating OR weight OR bmi OR body mass index OR calorie*) OR AB (((anorexi* OR bulimi* OR bing* OR purg*) W1 disorder*) OR ((eating OR feeding OR food) W1 (disorder* OR patholog*)) OR nutri* OR diet* OR eating OR weight OR bmi OR body mass index OR calorie*)

S8 S6 AND S7

S9 (MH "Qualitative Studies+") OR (MH "Multimethod Studies") OR (MH "Grounded Theory") OR (MH "Content Analysis") OR (MH "Practice Guidelines")

S10 TI qualitative OR AB qualitative

S11 S9 OR S10

S12 S5 AND S8 AND S11

**Supplementary Table 1.** List of included studies and their respective titles, main topics, and foci.

| **Study** | **Title** | **Main topic** | **Focus** |
| --- | --- | --- | --- |
| Ålgers  2012  *Finland* | Disordered eating and gender identity disorder: a qualitative study | Disordered eating | Trans Finnish adults' perspectives on eating and body image pathology in terms of presentation and causes, and the effect of gender transitioning on their relationship to food and body image |
| Brownstone  2022  *USA* | 'It’s just not comfortable to exist in a body': Transgender/gender nonbinary individuals’ experiences of body and eating distress during the COVID-19 pandemic | Disordered eating | Trans/nonbinary adults' perspectives on how COVID-19 affected their relationship to eating and body image |
| Cusack  2022  *USA* | “I’m still not sure if the eating disorder is a result of gender dysphoria”: Trans and nonbinary individuals’ descriptions of their eating and body concerns in relation to their gender | Disordered eating | Trans and nonbinary individuals' perceptions of the relationships between gender identity, gender expression, and weight and shape control behaviors |
| Duffy  2016  *USA* | Transgender clients' experiences of eating disorder treatment | Eating disorders | Trans and nonbinary adults diagnosed with eating disorders' experiences with eating disorders treatment |
| Gordon  2016  *USA* | “I have to constantly prove to myself, to people, that I fit the bill”: Perspectives on weight and shape control behaviors among low-income, ethnically diverse young transgender women | Disordered eating | Low-income, ethnically-diverse, young trans women with high HIV risk's perspectives on weight and shape control behaviors |
| Harrop  2023  *USA* | "How do I exist in this body...that's outside of the norm?" Trans and nonbinary experiences of conformity, coping, and connection in atypical anorexia | Eating disorders | Trans and nonbinary adults' experiences living with atypical anorexia in the context of a gender-diverse identity |
| Hartman-  Munick  2021  *USA* | Eating disorder screening and treatment experiences in transgender and gender diverse young adults | Eating disorders | Trans and gender-diverse young adults' experiences with and needs for eating disorders screening and treatment |
| Joy  2022  *Canada* | Exploring the influence of gender dysphoria in eating disorders among gender diverse individuals | Eating disorders | Gender-diverse Canadians' experiences accessing treatment for eating disorders |
| Kirby & Linde  2020  *USA* | Understanding the nutritional needs of gransgender and gender-nonconforming students at a large public Midwestern university | Nutritional needs | Trans and gender-nonconforming students' experiences of nutrition-related health disparities and barriers to adequate nutrition and health |
| Pham  2023  *USA* | Understanding the complex relationship between one's body, eating, exercise, and gender-affirming medical care among transgender and nonbinary adolescents and young adults | Disordered eating | Trans adolescents' and young adults' experiences of body, eating, and exercise, and their relationships to gender identity and gender-affirming medical care |
| Romito  2021  *USA* | Exploring transgender adolescents’ body image concerns and disordered eating: Semi-structured interviews with nine gender minority youth | Eating disorders | Trans adolescents' experiences of gender identity, gender transitioning, body image, and disordered eating |
| Russomanno 2019  *USA* | Food insecurity among transgender and gender nonconforming individuals in the southeast United States: A qualitative study | Food insecurity | Trans and gender non-conforming people's experiences of food insecurity |
| Schier & Linsenmeyer  2019  *Worldwide* | Nutrition-related messages shared among the online transgender community: A netnography of YouTube vloggers | Nutritional knowledge | Trans community members' food and nutrition messages shared via online vlogging |
| Zamantakis & Lackey  2022  *Worldwide* | Dying to be (a)gendered: An exploratory content analysis of trans/nonbinary people’s experiences with eating disorders | Eating disorders | Trans and nonbinary people's blog or vlog accounts of eating disorder experiences |

**Supplementary Material Table 2.** *Description of included studies’ sample characteristics and demographics.*

| **Study** | **Sample** | **Assigned Sex** | **Gender Identity** | **Age** | **Ethnic Identity** |
| --- | --- | --- | --- | --- | --- |
| Ålgers  2012  *Finland* | 20 Finnish trans adults | Female (55%)  Male (45%) | Trans men (55%)  Trans women (45%) | *M* = 35.20  *SD* = 12.10  Range = 21-62 | Breakdown not provided |
| Brownstone  2022  *USA* | 13 trans/nonbinary adults | Breakdown not provided | Nonbinary (76.92%)  Trans women (7.69%)  Nonbinary, trans men (7.69%)  Gender questioning, cisgender men (7.69%) | *M* = 31.24  Range = 19-53 | White (61.54%)  BIPOC (38.46) |
| Cusack  2022  *USA* | 82 trans/nonbinary individuals with self-reported eating and/or body image concerns | Breakdown not provided | Trans women (29.27%)  Nonbinary (26.83%)  Trans men (19.51%) | *M* = 24.65  *SD* = 5.32  Minimum = 18 | White (87.80%)  Hispanic/Latinx (4.88%)  Multiethnic (3.66%)  Prefer not to disclose (2.44%)  Asian (1.22%) |
| Duffy  2016  *USA* | 84 trans/gender-diverse adults with self- or professionally-diagnosed eating disorders | Breakdown not provided | Nonbinary (57%)  Trans men (36%)  Trans women (7%) | *M* = 22.3  *SD* = 3.91  Range = 18-33 | White (79.8%)  Multiethnic (11.9%)  Black (3.6%)  Hispanic/Latinx (3.6%)  Asian (1.2%) |
| Gordon  2016  *USA* | 21 trans women with (n = 16) and without (n = 5) experiences of disordered eating or weight control | Male (100%) | Trans women (100%) | *M* = 24  SD = 4.3  Range = 18-32 | Multiethnic (38.10%)  Black (19.05%)  Latina (19.05%)  White (19.05%)  Asian (4.76%) |
| Harrop  2023  *USA* | 9 trans/nonbinary adults with atypical anorexia | Female (88.89%)  Male (11.11%) | Nonbinary or genderqueer (77.78%)  Female (11.11%)  Male (11.11%) | *M* = 29.78  *SD* = 6.87  Range = 18-38 | White (77.78%)  African American, white (11.11%)  Hispanic, white (11.11%) |
| Hartman-  Munick  2021  *USA* | 32 trans/nonbinary adults living in the US who mentioned ED screening/treatment during a larger qualitative study | Breakdown not provided | Trans men (41%)  Nonbinary (28%)  Trans women (25%)  Another gender (6%) | 18-21 (31%)  22-25 (34.5%)  26-30 (34.5%)  Range = 18-30 | White (62%)  Hispanic/Latinx (13%)  Multiethnic (13%)  Asian (6%)  Black/African American (6%) |
| Joy  2022  *Canada* | 7 gender-diverse adults living in Canada who have accessed eating disordered treatment | Breakdown not provided | Nonbinary (42.86%)  Genderqueer (14.29%)  Trans woman (14.29%)  Nonbinary, trans women (14.29%)  Nonbinary, genderfluid (14.29%) | Range = 20-38 | White (85.71%)  Acadian (14.29%) |
| Kirby & Linde  2020  *USA* | 26 trans or gender-nonconforming students enrolled at a public Midwestern university | Female (96.15%)  Male (3.85%)  Intersex (0%) | Written-in gender identity (38.46%)  Trans males/men (26.92%)  Genderqueer/gender nonconforming (23.08%)  Male (7.69%)  Trans females/woman (3.85%) | *M* = 22.7  Minimum = 18 | White (73.33%)  Hispanic/Latinx (13.33%)  Asian/Pacific Islander (6.67%)  Black (3.33%)  Indigenous (3.33%) |
| Pham  2023  *USA* | 23 trans and nonbinary adolescents and young adults receiving services from a gender-affirming clinic | Female (47.8%)  Male (52.2%) | Transfeminine (44%)  Transmasculine (39%)  Nonbinary or genderfluid (17%) | *M* = 16.9  Range = 13-19 | White (82.6%)  Asian (8.7%)  Native American (8.7%) |
| Romito  2021  *USA* | 9 trans adolescents living in the US | Female (77.78%)  Male (22.22%) | *Participants endorsed multiple genders:*  Trans (19.23%)  Trans male (15.38%)  FtM (15.38%)  Trans female (7.69%)  Female (7.69%)  Genderqueer (7.69%)  Agender (7.69%)  MtF (3.85%)  Genderfluid (3.85%)  Female to male (3.85%)  Nonbinary (3.85%)  Male (3.85%) | *M* = 17  *SD* = 1.22  Range = 16-20 | White (77.78%)  Ethnic minority (22.22%) |
| Russomanno 2019  *USA* | 20 trans or gender non-conforming adults with self-reported experiences of food insecurity | Breakdown not provided | Trans male (40%)  Nonbinary (20%)  Trans female (15%)  Genderfluid (10%)  Genderqueer (10%)  Gender nonconforming (5%) | Range = 18-50 | Breakdown not provided |
| Schier & Linsenmeyer  2019  *Worldwide* | 30 vlogs by trans creators with nutrition- or food-related content | Female (63.3%)  Male (36.6%) | FtM (63.3%)  MtF (36.6%) | Breakdown not provided | Breakdown not provided |
| Zamantakis & Lackey  2022  *Worldwide* | 12 bloggers and 4 vloggers with content relating to eating disorders and being trans or nonbinary | Female (80%)  Male (20%) | Nonbinary AFAB (46.67%)  Trans men (33.33%)  Trans women (13.33%)  Nonbinary AMAB (6.67%) | Breakdown not provided | White (86.67%)  Southeast Asian (6.67%)  Multiethnic Asian (6.67%) |

*Note.* FtM = Female-to-male, MtF = Male-to-female, AFAB = assigned female at birth, AMAB = assigned male at birth

**Supplementary Material Table 3.** Themes and sub-themes based on 264 participant quotes extracted from the 14 studies.

| **Sub-Themes** | **# of**  **Articles** | **# of**  **Quotes** | **Example Quote** |
| --- | --- | --- | --- |
| **Theme 1: Lack of Resources or Access** | **10** | **49** |  |
| Poor nutrition due to financial insecurity | 5 | 30 | “[I was] eating really unhealthy food, because it was cheaper. Gained weight, and felt sick and tired all the time. Your quality of life really suffers when you put unhealthy things into your body” (19 p. 95) |
| Nutrition difficulties due to barriers to gender-affirming care | 5 | 5 | “My eating disorder started as a way to stop [menstruation] and breast development, so access to binders, hormone blockers/HRT [hormone replacement therapy] and gender affirming [care] would have helped me more when I was younger than therapy surrounding body image” (11 p. 4) |
| Weight as a barrier to gender-affirming medical interventions | 3 | 6 | “I'm pursuing bottom surgery (phalloplasty), and I'm required to lose weight for this…So this surgery that I've poured literally hundreds of hours into research and insurance appeals, money into traveling out of state…could literally be for nothing if I don't lose 10-15 pounds. It's a small amount of weight but coming from a disordered eating background, it's so hard not to fall back on my former restrictive patterns” (11 p. 3) |
| Nutrition myths | 1 | 8 | “I ended up bingeing in the kitchen late last night but I still tracked everything...this is to be expected when you’re cutting such a high deficit, like, you’re probably going to binge eventually. It’s just a psychological fact unless you’re extremely dedicated, binges are inevitable so you should plan for them and learn to control your binge and just psychologically rethink the way you think about a binge” (20 p. 345) |
| **Theme 2: Experiences with Care Providers** | **9** | **43** |  |
| Recommendations for care providers | 7 | 12 | “Recognize that being trans doesn’t necessarily cause an eating disorder but does inform how that eating disorder is experienced. This validation and competency leads to blossoming of sense of self and strength in who people are as a transgender/gay/lesbian/bisexual/asexual/queer and that strength can make all the difference” (21 p. 879) |
| Lack of trans literacy from care providers | 4 | 5 | “I felt that [gender presentation] was a conversation that they didn’t know how to hold space for [and they didn’t know] why it would matter so much to me…there’s no space to talk about how gender identity or identity in general fits within the space of EDs” (6 p. 11) |
| Avoiding possible transphobic experiences with care providers | 3 | 7 | “...definitely avoided, prolonged and suffered more because it just didn't feel safe (accessing treatment). I needed that medical safety, but I didn't have emotional safety” (3 p. 394) |
| Insensitivity from care providers | 3 | 7 | “My therapist asked me questions about my body, such as if I’d taken hormones or had ”the surgery" yet. She twice asked me if I was “really a boy or a girl,” despite my birth name and sex being on my records” (14 p. 142) |
| Erasure of gender identity by care providers | 2 | 7 | “I have been regularly misgendered over the course ofmy treatment. It’s kind of par for the course by now, but it’s still really upsetting. Even my therapist doesn’t really understand how to use my pronouns, and has referred to me as “girl" and “daughter,” despite the fact I’ve come out to her” (14 p. 142) |
| Having to educate care providers | 2 | 4 | “I am simply not interested in educating professionals about my gender or identity; it’s the one space where I do not have the energy left to do so” (14 p. 143) |
| Affirming care experiences | 2 | 3 | “When I went to treatment, my team used my real name (not my deadname), used my pronouns, and were open to learning about my gender. They even installed a gender-neutral bathroom sign! Now, it's helpful for me to have a clinician who doesn't judge me, who accepts me for who I am” (11 p. 4) |
| Lack of representation among care providers | 1 | 3 | “[An absence of diverse providers] is part of why I haven’t…pursued any sort of specialized [ED] treatment” (6 p. 11) |
| **Theme 3: Unique Challenges Faced by Gender-Diverse People** | **8** | **40** |  |
| Trans-specific ED recovery challenges | 6 | 17 | “In the trans community, your body is wrong. Like the gender that you know you are, that is you, and the body you’re in, if it doesn’t match what you think your body should look like, then you should change it. Whereas, the message on the recovery side is that your body is perfect the way it is and you need to accept it for what it is, and that is impossible for someone who experiences dysphoria because your body just doesn’t match who you know you are...Eating disorders are not the right way to go about eliminating your dysphoria but it does help honestly” (21 p. 881) |
| No acknowledgment of gender diversity in ED spaces | 5 | 14 | “Eating disorders are so heavily associated with cis, het, white women. It can make it harder for people who aren't that to recognise or to have other people who care about them recognise it” (3 p. 392) |
| No acknowledgment of EDs in gender diversity spaces | 4 | 12 | “It's really hard when you're someone in recovery to ﬁnd a space that's not just going to be a bunch of like White ladies .. . on the other side though someone who's nonbinary and someone who is in recovery, I don't quite identify with other trans masculine people because a lot of like FTM or trans masculine people are hyper ﬁxated in diet culture and hyper ﬁxated and having these buff “muscle-y” bodies” (22 p. 439) |
| **Theme 4: Gender Affirmation and Recovery** | **8** | **36** |  |
| Improved relationship with nutrition post-gender affirmation efforts | 7 | 21 | “After my breast reduction surgery I gained some weight, but I was only happy about it. I could imagine being a fat man, but I could never be a fat woman. There is a huge difference” (9 p. 308) |
| Concerns about nutrition/body post-gender affirmation efforts | 4 | 8 | “But also the [feminizing] changes can be difﬁcult in some ways just because of preexisting eating and body issues...as my dysphoria issues are starting to become less prominent...it is just making my sort of body image issues more—In terms of the weight and stuff...more prominent...overall, I’m probably in a better place, but...it’s just now that that’s sort of what I’m focusing on, I think” (13 p. 154) |
| Concerns about gender after ED recovery | 2 | 5 | “After discharge, I continued treatment through regular outpatient appointments and therapy sessions. What the doctors called “progress” was to me the methodical undoing months of my hard work. I could actually feel my body changing, feel myself sliding away from where I wanted to be. Gender was size, and size was gender” (21 p. 886) |
| Improved relationship with gender after ED recovery | 1 | 2 | “Only now that I have been more committed to recovery have I allowed myself the space to explore gender and the other options for bringing my body into alignment with my internal sense of gender” (10 p. 7) |
| **Theme 5: Disordered Eating to Conform with Social and Gender Norms** | **8** | **34** |  |
| Achieving normative gender ideals | 7 | 23 | “My disordered eating is directly related to how I feel I have to make myself look in order to be a “real” woman. I feel that I have to follow feminine beauty standards more strictly than others otherwise my gender will be called into question and as such I require myself to be quite thin” (10 p. 7) |
| Resisting gender norms | 3 | 8 | “...to stop attempting to ﬁt into that ‘woman box’, of what a perfect woman looks like, has felt like a big part of healing my relationship with food and movement and my body” (6 p. 10) |
| Fitting in with society | 2 | 3 | “[My eating problems are also related to the fact that] I have felt like an outsider ever since I was little. I have felt inadequate, like I don’t belong to the group, and because of that any criticism about what was most essential to me, my body and how desirable I am, was a really serious thing to me” (9 p. 307) |
| **Theme 6: Restrictive Nutrition as a “DIY” Solution to Gender Affirmation** | **10** | **33** |  |
| Manipulating secondary sex characteristics | 8 | 23 | "I have a false hope that by losing weight I will make my traditionally masculine features appear more feminine" (10 p. 5) |
| Feeling like one's identified gender | 6 | 9 | “[After losing a lot of weight] I could buy pants at the men’s department, and they ﬁt in a certain way, the right way, as I see it. And also, I felt strong, which I perceived as masculine” (9 p. 307) |
| Gender affirmation as motivation for ED recovery | 1 | 1 | “I was too unhealthy to keep on going the way I was, and it made me realize that if I ever wanted to start HRT [hormone replacement therapy], I had to be healthier than I was being” (14 p. 141) |
| **Theme 7: Exerting Control Over the Body** | **8** | **14** |  |
| Control | 6 | 11 | “My eating disorder allowed me to control the parts of me I was too scared to show the world, the parts that were too masculine to exist in a feminine body” (21 p. 878) |
| Shame | 2 | 2 | “Gender dysphoria greatly inﬂuences my body image and sense of self. I also have a lot of internalized homophobia/transphobia that sometimes creates shame and feelings that I don't deserve to eat” (10 p. 6) |
| Self-punishment | 1 | 1 | “I can't pass for what I am like, because there is no passing for something that so many people don't even know exists, don't even believe exists for something that doesn't have like a specific marker in broad society and it makes me feel like punishing my body and sometimes that leads to restricting sometimes that leads to bingeing sometimes it leads to over exercising” (22 p. 440) |
| **Theme 8: Coping with Gender Dysphoria** | **6** | **8** | “If you’re concentrating more on like, you know, ‘I might faint,’ you’re less concentrated on, like, ‘Everyone is viewing me as a girl, and that is painful” (12 p. 55) |
| **Theme 9: At the Intersection of Gender Diversity and Eating Disorders** | **5** | **7** | "It’s difficult in general for me to find therapy and help that understands me being trans, being a rape survivor, and having an eating disorder. I tend to find any help I seek for one, or any combination of those, fails in at least one of those areas” (14 p. 142) |
